# Metastatic gastric cancer presenting with shoulder-hand syndrome: a case report

**DOI:** 10.1186/1752-1947-2-240

**Published:** 2008-07-24

**Authors:** Marco Massarotti, Gianluigi Ciocia, Roberto Ceriani, Arturo Chiti, Bianca Marasini

**Affiliations:** 1 Rheumatology Unit, IRCCS Humanitas Clinical Institute, University of Milan, Via Manzoni, 20089 Rozzano, Milan, Italy; 2 Nuclear Medicine Unit, IRCCS Humanitas Clinical Institute, Via Manzoni, 20089 Rozzano, Milan, Italy; 3 Internal Medicine and Hepatology Unit, IRCCS Humanitas Clinical Institute, University of Milan, Via Manzoni, 20089 Rozzano, Milan, Italy

## Abstract

**Introduction:**

Shoulder-hand syndrome is a relatively rare clinical entity classified as a complex regional pain syndrome type 1 and consisting essentially of a painful 'frozen shoulder' with disability, swelling, vasomotor or dystrophic changes in the homolateral hand. The pathophysiology is not completely clear but a predominant 'sympathetic' factor affecting the neural and vascular supply to the affected parts seems to be involved. Shoulder-hand syndrome has been related to many surgical, orthopedic, neurological and medical conditions; it is more often seen after myocardial infarction, hemiplegia and painful conditions of neck and shoulder, such as trauma, tumors, cervical discogenic or intraforaminal diseases and shoulder calcific tendinopathy, but has also been associated with herpetic infections, brain and lung tumors, thoracoplasty and drugs including phenobarbitone and isoniazid. The diagnosis of shoulder-hand syndrome is primarily clinical, but imaging studies, particularly bone scintigraphy, may be useful to exclude other disorders.

**Case presentation:**

We report the case of a 67-year-old woman who presented with shoulder-hand syndrome as the initial manifestation of gastric cancer which had metastasized to bone.

**Conclusion:**

Wider investigations are advisable in patients with atypical shoulder-hand syndrome. To the best of the authors' knowledge this is the first case of shoulder-hand syndrome associated with metastatic gastric cancer.

## Introduction

Shoulder-hand syndrome (SHS) is a relatively rare clinical entity classified as a complex regional pain syndrome type 1 (CRPS1), indicated previously as reflex sympathetic dystrophy (RSD) [1]. SHS consists essentially of a painful 'frozen shoulder' with disability, swelling, vasomotor or dystrophic changes in the homolateral hand. The shoulder involvement usually precedes, sometimes accompanies or rarely follows the changes in the hand. This syndrome, which was first described in the 1950s [2], is the most extensive CRPS1 affecting upper extremities. The pathophysiology is not completely clear but a predominant 'sympathetic' factor affecting the neural and vascular supply to the affected parts seems to be involved.

## Case presentation

We report the case of a 67-year-old woman who presented to our institute in August 2006 with a 2-month history of pain and swelling of the right hand and wrist, and a more recent onset of pain in the right shoulder. Her medical history included arterial hypertension and depression. Blood tests performed prior to hospitalization were unremarkable except for a high erythrocyte sedimentation rate (ESR) of 78 mm/hour. Plain radiography of the right hand showed osteoarthritis of the first carpometacarpal joint. Non-steroidal anti-inflammatory drugs and acetaminophen were given without significant improvement.

**Figure 1:**
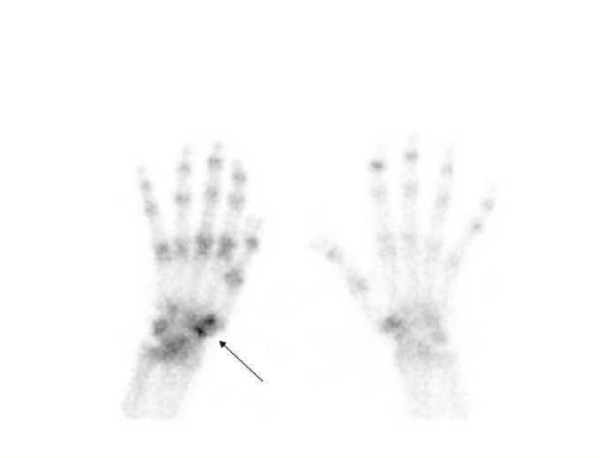
**Late phase bone scintigraphy of the hand and wrist**. 3 hours after injection planar ventral view demonstrated light uptake at the carpo-metacarpal joint of the right hand.

**Figure 2:**
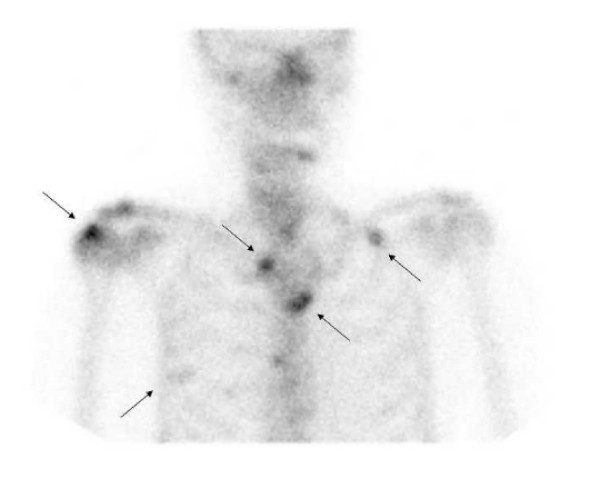
**Scintigraphy of the chest**. 3 hours after inlìjection planar antero-posterior view demonstrated diffuse spots of hyperfixation in the right humeral head and acromion, medial right clavicle, sternum and ribs. Other views confirmed bone localization of metastases on more sites.

On physical examination, the fingers of the right hand were flexed, the right wrist was swollen and the right shoulder was extremely painful with a limited range of motion. Routine blood tests were normal but ESR was still high (46 mm/hour). A radiograph of the right shoulder showed demineralization of the humeral head and of the scapula, and an ultrasound study of the right shoulder, wrist and hand showed a supraspinatus tendinopathy without tendon tears and swelling of radiocarpal and intercarpal joints with marked power Doppler signal. SHS was suspected and a radionuclide scintigraphy was performed (Figures 1 and 2). The triphasic study of the right arm revealed an increased perfusion with increased and delayed activity of bone images, suggesting RSD of the wrist.

However, the whole body study, which revealed diffuse spots of hyperfixation in the right humeral head and acromion, medial right clavicle, sternum, ribs, dorsal and lumbar spine and pelvis, was consistent with skeletal metastases. Magnetic resonance imaging (MRI) of the spine and pelvis confirmed the presence of multiple metastases located in the dorsal and lumbar spine, sacrum, pelvis and both femurs. The patient was treated with a single intravenous infusion of 90 mg pamidronate.

Further diagnostic studies were performed to identify the primary neoplasm. Mammography, thyroid ultrasound and lung computed tomography scan were unremarkable. Gastric endoscopy revealed an adenocarcinoma of the angular region. Despite chemotherapy, a radionuclide scan performed in November 2006 showed progression of the metastatic bone lesions. Signs and symptoms of SHS were completely resolved, but the images of the right hand were unmodified.

## Discussion

SHS has been found in association with a number of surgical, orthopedic, neurological and medical conditions. It is more often seen after myocardial infarction, hemiplegia and painful conditions of neck and shoulder, such as trauma, tumors, cervical discogenic or intraforaminal diseases and shoulder calcific tendinopathy, but has also been associated with herpetic infections, brain and lung tumors, thoracoplasty and drugs such as phenobarbitone and isoniazid [2-6].

The diagnosis of SHS is primarily clinical. Blood tests, including ESR, are normal and no specific antigens or antibodies are found [7]. Imaging studies have been used mainly to exclude other disorders. Plain radiography reveals demineralization, probably related to lack of use [8]; MRI demonstrates peri-articular marrow edema, soft tissue swelling and joint effusions [8]; bone scintigraphy shows increased peri-articular activity in the affected limb and is more sensitive than plain radiography [9]. Rapid evaluation is necessary, because the earlier treatment is started, the better the prognosis [8]. Many approaches have been proposed for RSD treatment, such as short-term glucocorticoids, ketamine, muscle relaxants, benzodiazepines, antidepressants, anticonvulsants, ketanserin, opioids, intensive physical therapy, nerve blocks, sympathectomy, intraspinally administered drugs and neuromodulatory therapies [10,11], and the efficacy of bisphosphonates has been documented in several trials [10-12].

## Conclusion

Wider investigations are advisable in patients with atypical SHS, including those with indications such as increased inflammation markers, as were present in this patient. To the best of the authors' knowledge this is the first reported case of SHS associated with metastatic gastric cancer.

## Abbreviations

CRPS1: complex regional pain syndrome type 1; ESR: erythrocyte sedimentation rate; MRI: magnetic resonance imaging; RSD: reflex sympathetic dystrophy; SHS: shoulder-hand syndrome.

## Competing interests

The authors declare that they have no competing interests.

## Authors' contributions

All authors contributed equally to this case report. All authors read and approved the final manuscript.

## Consent

Written informed consent was obtained from the patient for publication of this case report and accompanying images. A copy of the written consent is available for review by the Editor-in-Chief of this journal.
